# Editorial: Novel treatments for cardiovascular diseases by targeting inflammation, oxidative stress, and cell death

**DOI:** 10.3389/fcvm.2024.1538222

**Published:** 2025-01-07

**Authors:** Dong Fan, Li Wang, Abhijit Chakraborty, Cheng-Lin Zhang, Feng Hua Yang

**Affiliations:** ^1^ Department of Pathophysiology, Zhuhai Campus of Zunyi Medical University, Zhuhai, China; ^2^ Department of Biomedical Sciences, City University of Hong Kong, Hong Kong, Hong Kong SAR, China; ^3^ Department of Investigational Cancer Therapeutics, The University of Texas MD Anderson Cancer Center, Houston, TX, United States; ^4^ Department of Pathophysiology, Shenzhen University Medical School, Shenzhen, China; ^5^ Guangdong Provincial Biotechnology Research Institute, Guangdong Provincial Laboratory Animals Monitoring Center, Guangzhou, China

**Keywords:** cardiovascular disease, oxidative stress, inflammation, cell death, therapeutic targets

**Editorial on the Research Topic**
Novel treatments for cardiovascular diseases by targeting inflammation, oxidative stress, and cell death

Cardiovascular diseases (CVDs) remain the leading causes of morbidity and mortality worldwide, representing a significant burden on global public health systems (1). The pathogenesis of CVDs is complex, with inflammation, oxidative stress, and cell death playing critical roles, making them promising targets for therapeutic intervention (2–5), such as anti-inflammatory therapies for atherosclerotic CVDs with colchicine or an antibody against interleukin 1β (IL-1β) (6–9). The collection features four comprehensive articles that explore the multifaceted nature of CVDs, each offering new insights into how inflammation, oxidative stress, and cell death contribute to various CVDs. Topics addressed include vascular complications in diabetes, myocardial injury caused by ischemia-reperfusion (I/R) injury, sepsis, and calcific aortic valve disease (CAVD), as well as some potential markers and therapeutic approaches.

The review by Yang et al. categorizes diabetic vascular complications into macrovascular (e.g., diabetic atherosclerosis, coronary artery disease, and peripheral vascular diseases) and microvascular (e.g., diabetic nephropathy, retinopathy) diseases, which are key contributors to the high morbidity and mortality rates in individuals with diabetes. Besides diabetic vascular complications, I/R, sepsis, and CAVD can also lead to myocardial damage. Myocardial infarction (MI) is a macrovascular disease primarily caused by atherosclerosis and an occlusive thrombus within a coronary artery (10, 11). I/R injury can occur at the reperfusion therapy after MI and is related to cardiac dysfunction, arrhythmias, and pathological cardiac remodeling (4, 12, 13). Septic cardiomyopathy (SCM) is a critical but reversible condition in intensive care units where cardiac dysfunction results from a dysregulated host response to infection and microvascular dysfunction (14–16). CAVD is a common valvular disease with characteristic of aortic valve fibrosis and calcification, and can progress into aortic stenosis with a high mortality rate (17).

Inflammation, oxidative stress, and cell death are enhanced in diabetic CVDs, I/R injury, SCM, and CAVD, and they also result in progress of these CVDs (3–5, 14, 17–19). Metformin, dipeptidyl peptidase-4 inhibitors (DPP-4i), and sodium-glucose cotransporter 2 inhibitors (SGLT2i) have been utilized to treat diabetes and can protect endothelial cells through reducing inflammation and the production of reactive oxygen species (ROS), leading to amelioration of diabetic vascular diseases. Meanwhile, these therapeutic strategies have some side effects to be assessed when they are employed, such as nausea and abdominal pain with metformin, an enhanced risk of heart failure with DPP-4i, and urinary tract infection with SGLT2i. Some novel therapeutic approaches, such as microRNAs and stem cell therapies, are also potential ways to improve endothelial cell function by inhibiting oxidative stress and cell death, but they demand further investigation to combat the limitations of instability, off-target effects, and immune responses.

The treating effects for CVDs can vary with different species. The study by Haugsten Hansen et al. on a large animal I/R model proves that oxidative stress was only enhanced in early reperfusion, but systemic antioxidative treatment with N-acetylcysteine (NAC) did not reduce the oxidative stress or protect against I/R-induced arrhythmias. The results suggest that while oxidative stress is a key player in MI and I/R injury, broad-spectrum antioxidative therapies may not be effective. This underscores the need for more targeted therapies and a deeper understanding of ROS signaling especially in large mammalian I/R models.

The study by Chen et al. explores the effects of low molecular weight heparin (LMWH) on heparanase levels in patients with SCM. Their findings reveal that LMWH treatment leads to a significant reduction in heparanase and syndecan-1 levels, alongside a decrease in inflammatory markers and an enhancement in cardiac function and microcirculation. Specifically, LMWH inhibited oxidative stress and mitigated heart damage, thereby improving clinical outcomes for SCM patients. The study posits that heparanase may serve as a diagnostic marker and a potential therapeutic target for SCM, suggesting a novel approach to managing this condition. Furthermore, the reduction in lactate levels post-treatment indicates improved tissue perfusion, reinforcing the therapeutic efficacy of LMWH in enhancing patient prognosis. Overall, the research underscores the multifaceted benefits of LMWH in SCM management, highlighting its role in reducing inflammation and protecting cardiac function.

Therefore, a good diagnostic marker can facilitate the diagnosis of a disease, and it can also be a therapeutic target. The study by Yu et al. screened potential diagnostic biomarkers for CAVD by leveraging publicly available gene expression datasets from the Gene Expression Omnibus (GEO) database. Seventeen pyroptosis-related differentially expressed genes (DEGs) were identified between calcified and normal aortic valve tissues. Among these DEGs, three genes (TREM1, TNFRSF11B, and PGF) were significantly increased in the CAVD tissues, which were further identified as the diagnostic candidate genes for CAVD by machine learning techniques, namely least absolute shrinkage and selection operator (LASSO) regression and random forest. The identified genes, TREM1, TNFRSF11B, and PGF, were subsequently validated using independent datasets and showed good diagnostic performance. CIBERSORT and Pearson correlation analysis further elucidate a positive correlation between the expression levels of these three identified biomarkers and the proportions of some pro-inflammatory immune cell types. Integrating these methods in studying pyroptosis and immune responses in CAVD underscores their potential therapeutic value, as these genes could serve as targets for novel treatments.

Together, these articles highlight the multifaceted nature of CVDs, emphasizing the critical roles of inflammation, oxidative stress, and endothelial dysfunction in various cardiovascular conditions. They enhance our understanding of the complex interplay between inflammation, oxidative stress, and cell death, while demonstrating the potential for improving outcomes through the reduction of inflammatory markers and the enhancement of cardiac function. This collection also underscores the need for targeted therapies focused on specific aspects of ROS signaling. Furthermore, this collection highlights the importance of personalized approaches by using machine learning to analyze gene expression data, enabling the identification of biomarkers and potential therapeutic targets for CVDs. By offering deeper insights into the molecular and cellular mechanisms driving CVDs, as well as innovative research tools, this collection contributes to ongoing efforts to develop more effective, personalized treatments that can alleviate the global burden of CVDs and improve long-term cardiovascular health.
